# A meta-analysis on neural changes of cognitive training for mental disorders in executive function tasks: increase or decrease brain activation?

**DOI:** 10.1186/s12888-022-03796-4

**Published:** 2022-03-01

**Authors:** Jin Yang Li, Huiqin Wu, Shiting Yuan, Chun Wang, Qian Wang, Yuan Zhong, Ning Zhang, Kathi Heffner, Peter T. Fox

**Affiliations:** 1 Nan jing Brain Hospital affiliated to Nanjing Medical University, Nanjing, 210029 Jiangsu China; 2 Functional Brain Imaging Institute of Nanjing Medical University, Nanjing, 210029 Jiangsu China; 3 BeiJing TianTan Hospital, Capital Medical University, Beijing, 100050 China; 4 School of Psychology, Nanjing Normal University, Nanjing, 210029 Jiangsu China; 5 Department of Psychiatry, University of Rochester School of Nursing, Rochester, New York 14622 USA; 6 South Texas Veterans Healthcare System, University of Texas Health San Antonio, San Antonio, USA; 7 Research Imaging Institute, University of Texas Health San Antonio, San Antonio, USA

**Keywords:** Cognitive training, Mental disorder, Neuroimaging, Meta-analysis

## Abstract

**Background:**

Cognitive impairment is often found in patients with psychiatric disorders, and cognitive training (CT) has been shown to help these patients. To better understand the mechanisms of CT, many neuroimaging studies have investigated the neural changes associated with it. However, the results of those studies have been inconsistent, making it difficult to draw conclusions from the literature. Therefore, the objective of this meta-analysis was to identify consistent patterns in the literature of neural changes associated with CT for psychiatric disorders.

**Methods:**

We searched for cognitive training imaging studies in PubMed, Cochrane library, Scopus, and ProQuest electronic databases. We conducted an activation likelihood estimation (ALE) for coordinate-based meta-analysis of neuroimaging studies, conduct behavioral analysis of brain regions identified by ALE analysis, conduct behavioral analysis of brain regions identified by ALE analysis, and then created a functional meta-analytic connectivity model (fMACM) of the resulting regions.

**Results:**

Results showed that CT studies consistently reported increased activation in the left inferior frontal gyrus (IFG) and decreased activation in the left precuneus and cuneus from pre- to post- CT.

**Conclusion:**

CT improves cognitive function by supporting language and memory function, and reducing neuronal resources associated with basic visual processing.

## Background

The prevalence and disease burden of psychiatric disorders have remained incredibly high globally, with a total prevalence rate of about 30.5% [1]. Common to many psychiatric disorders is a substantial decline in cognitive function [2]. Cognitive training (CT), also known as cognitive remediation or cognitive remediation therapy, includes interventions in which patients repeatedly perform cognitive tasks in order to improve their cognitive abilities [3]. CT is an effective means of improving neuropsychological deficits in many different populations [4, 5].

The effects of CT on brain function have been studied extensively using neuroimaging techniques. Neuroimaging studies indicate that CT is associated with structural and functional alterations [6–8]. Most functional neuroimaging studies of CT focus on executive function. Executive functions are a collection of cognitive processes that help us to regulate our thoughts and behaviours to make plans, solve problems, and attain goals [9]. Major components of executive functioning include attention, inhibition, self-regulation, working memory, cognitive flexibility, planning, organization, problem-solving, and performance-monitoring [10]. A series of studies have shown that CT can improve executive function in patients with psychiatric disorders [11–13]. However, these studies do not always report concordant results, making it difficult to draw conclusions about the neural changes associated with CT. Recently, some meta-analysis and review suggests that cognitive training can improve the cognitive function of schizophrenia by activating the frontal brain regions [14, 15].

To infer a reliable conclusion from disparate results requires appropriate statistical methods. Meta-analysis is one method used by researchers to objectively identify reliable effects across the literature by pooling results across many studies to test for a significant convergence of findings [16]. Meta-analytic techniques can be used to build models and detect emergent properties of neural systems through large-scale data mining and computational modeling [17].

The objective of the present study was to use meta-analyses to identify neural changes associated with CT that are common across psychiatric disorders. Despite different neuropathology that may be a part of a specific disorder, it is important to understand common effects of CT on neural circuitry. If the results of this meta-analysis show both increased and decreased brain activation, we can infer that CT improves cognitive function through more than one pathway. Furthermore, the current study analyzed functional interactions between regions implicated in the meta-analysis, thereby identifying brain networks that are modified following CT, and providing clues regarding its mechanism of action.

## Methods

### Literature search and selection

We searched for studies from electronic databases including PubMed, Cochrane library, Scopus, and ProQuest. We used the following search criteria: Title/Abstract “cognitive training or cognitive remediation or cognitive rehabilitation or cognitive stimulation” and “MRI or magnetic resonance imaging or fMRI or functional magnetic resonance imaging or PET or positron emission tomography or SPECT or single photon emission computed /tomography”. Reviews were excluded.

**Fig. 1:**
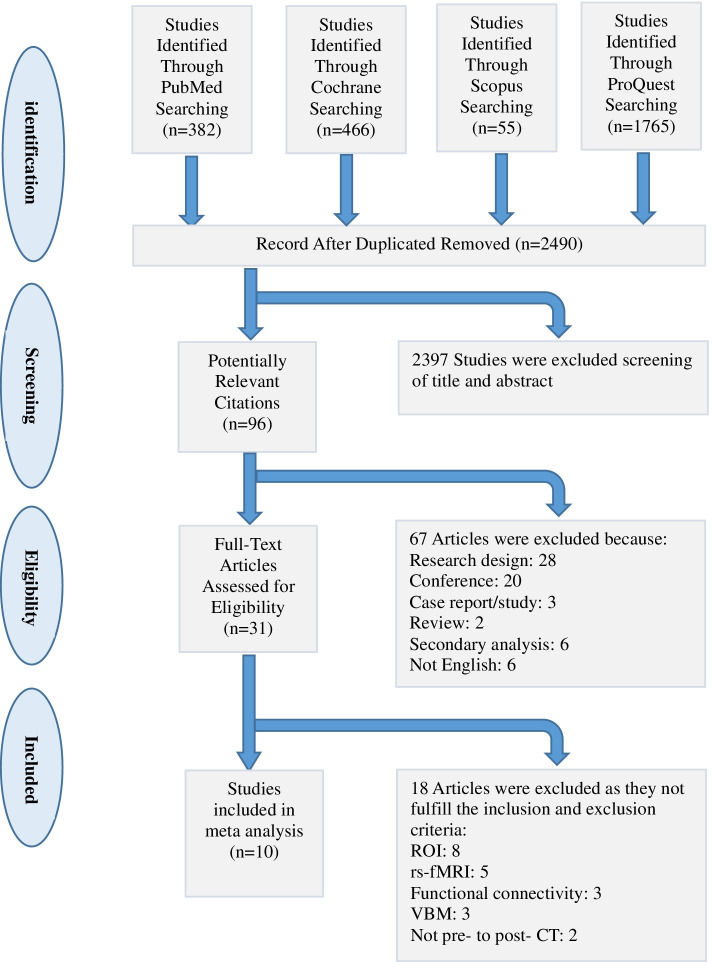
Flowchart of the Different Steps Conducted. In total, 16 studies remained. rs - fMRI = Resting-State functional MRI; FC = functional connectivity; VBM = voxel-based morphometric; ROI = region of interest

For the papers resulting from this search, we applied the following inclusion criteria [18]: (1) Participants included patients with a diagnosed psychiatric disorder (2) The paper was a study of executive function (3) A task was presented during image acquisition (4) The analysis focused on longitudinal changes in brain activation from pre- to post- CT (5) The analysis was a whole-brain voxel-wise analysis (6) The results were reported using stereotactic coordinates (Talairach/MNI) (x, y, z). The following exclusion criteria were applied: (1) Newspaper reports or abstract publications (2) Studies that were not in English (3) Resting-state fMRI, functional connectivity, voxel-based morphometry, or Region-of-Interest analyses. The entire search process is shown in the flowchart below (Fig. 1).

After applying these criteria, the resulting articles were coded into the BrainMap database [19] according to paradigm and direction of effect. The locations of the maximum voxel in each cluster were reported in Montreal Neuroimaging Institute (MNI) space. Studies reporting foci in Talairach space were converted to MNI space using the “Talairach to SPM” conversion function implemented in Ginger ALE.

### Overlay analysis

To identify whether the regions activated by different tasks overlap, we performed a coordinate-based meta-analyses of executive functioning using the anatomic likelihood estimation (ALE) method per each task and completed within-group meta-analyses for each BrainMap domain to find domain-specific patterns of activation [20]. For the overlay analysis, we choose cluster-level of *p* = 0.05, minimum volume = 200, and threshold permutations = 1000.

### ALE Meta-analysis

An ALE approach for coordinate-based meta-analysis was used to assess the overlap of foci reported in the individual studies by modeling them as 3-dimensional Gaussian probability distributions centered at the respective coordinates. The ALE method accommodates the spatial uncertainty associated with each single focus [21].

For brain areas reported with increased or decreased activations, two ALE meta-analyses were performed separately using *P* value = 0.05, cluster-level = 0.05 and threshold permutations =1000. First, a modeled activation map was created from the probabilities of all foci at each voxel. Then, a voxel-wise ALE map was created from the union of all modeled activation maps. The ALE map indicates the degree of convergence of results at each particular location in the brain [22]. A voxel-level FWE for meta-analyses via the ALE method was used for significance. The studies included in the map of increased activation to CT included 9 experiments in 8 papers reporting 37 foci. The studies included in the map of decreased activation to CT included 6 experiments in 5 papers with 27 foci.

### Behavior analysis

In order to determine the behavior associated with each region within the same network, it is important to explicate the functional associations of individual regions across a wider anatomical scope that spans various networks. We used a plug-in software “Behavior Analysis” in the BrainMap database to map the functional correlates of behavioral phenotypes across regions. This method is based on the “Behavior Domain” describing behaviors and psychological processes. The behavior domain mainly includes cognition, action, perception, emotion, and internal concepts. When Z-score > 3, the behavioral domain was considered to be significantly associated with the activated brain region.

### Functional Meta-analytic connectivity modeling (fMACM) procedure

Meta-analytic connectivity modeling (MACM) was used to identify functional co-activation of regions resulting from the ALE meta-analysis. MACM assesses the brain-wide co-activation patterns across a large number of functional neuroimaging results and identifies areas that show consistent co-activation with a seed region [23, 24]. For the current paper, we limited our search to fMACM (fMRI and PET analyses) in order to find task-dependent functional co-activations. Our seed regions included the 3 locations identified in our ALE analysis (left inferior frontal gyrus (IFG), left precuneus, left cuneus). The ROIs were obtained by drawing a circle with a radius of 9 around the maximum point of activation. We set *p* value = 0.05.

## Results

### Literature search results

10 articles met all criteria and were coded into the BrainMap database according to paradigm and direction of effect. Characteristics of the 10 included studies are shown in Table 1. Of the 10 included studies, there were 6 schizophrenia, 2 anorexia nervosa, 1 attention deficit hyperactivity disorder, and 1 mood disorder. Participants in these studies were aged from 11 to 41, including both patient and control groups. The studies involved 5 different executive function tasks: n-back, reasoning / problem solving, task switching, Go/No-Go, and visuospatial attention.

**Table 1:** Characteristics of the 16 induced studies

Author	Disorder	Patients/n	control patients/n	Healthy/n	Mean age	Gender (male/female)	Intervention	Cognition	Imaging Task	Sessions
Wykes, 2002 [25]	SCZ	3	6	6	35	18/0	CRT	Working Memory	N-back	12 weeks
Brockmeyer, 2016 [26]	anorexia nervosa	12	12	0	22.82	–	CRT	Cognitive flexibility	task switching	30 sessions over 3 week
Subramaniam, 2014 [27]	SCZ	16	14	15	40.69	12/4	CT	Working Memory	N-back	80 h(16 weeks)
Vianin, 2014 [11]	SCZ	8	8	0	27.63	6/2	CRT	Executive function	Problem Solving	14 weeks
Fonville, 2014 [28]	anorexia nervosa	9	0	9	22	–	CRT	Executive function	attention	10 sessions over 2 months
Penades, 2013 [29]	SCZ	17	18	15	36.35	12/5	CRT	Working Memory	N-back	40 sessions over 4 months
Meusel, 2013 [30]	mood disorder	35	0	15	39. 7	7/28	CR	Working Memory	N-back	10 weeks
Bor, 2011 [31]	SCZ	8	9	15	30.5	6/2	CRT	Working Memory	N-back	7 weeks
Hoekzema, 2010 [32]	ADHD	9	10	0	11.22	8/1	CT	Attention	problem solving, Go/No-Go	10 days
Rowland, 2010 [33]	SCZ	17	0	17	31.4	9/8	learning training	Relational learning	Visuospatial attention	1 session

Note. SCZ, schizophrenia(F20.9); ADHD, Attention Deficit/Hyperactivity Disorder(F90)

### Overlay analysis of task-specific maps

Five coordinate-based meta-analyses of executive functioning were performed using the ALE method for each executive function imaging task. Table 2 lists the studies included in each task-wise map. According to the results, n-back activated right Mid Frontal Gyrus (BA6), left Cingulate Gyrus (BA32), left Inf Parietal Lobule (BA40), right Mid Frontal Gyrus (BA9), right Inf Parietal Lobule (BA40), left Precentral Gyrus (BA9), left Sub-Gyrus (BA6), and right Precuneus (BA7); Reasoning activated right Insula (BA13), right Inf Frontal Gyrus (BA9), left Sup Parietal Lobule (BA7), right Sup Parietal Lobule (BA7), and right Precuneus (BA31); Task switching activated left Sup Frontal Gyrus (BA6), right Insula (BA13), left Inf Parietal Lobule (BA40), left Precentral Gyrus (BA6); Go-NoGo activated left Med Frontal Gyrus (BA6) and right Med Frontal Gyrus (BA6); and visuospatial attention activated left Sup Parietal Lobule (BA7), right Precuneus (BA7), and left Med Frontal Gyrus (BA6).

**Table 2:** Studies from the BrainMap database for the 5 executive functioning tasks reported in the included publications

Tasks	Papers	Subjects	Experiments	Locations
1. N-back	45	748	153	1282
2. Reasoning/Problem Solving	45	1158	207	1607
3. Task switching	45	927	166	1332
4. Go/No-Go	45	951	149	1577
5. Visuospatial attention	45	519	111	1303

Note. 45 papers were used per analysis is because it is the lowest common number of papers among the 5 tasks

**Fig. 2:**
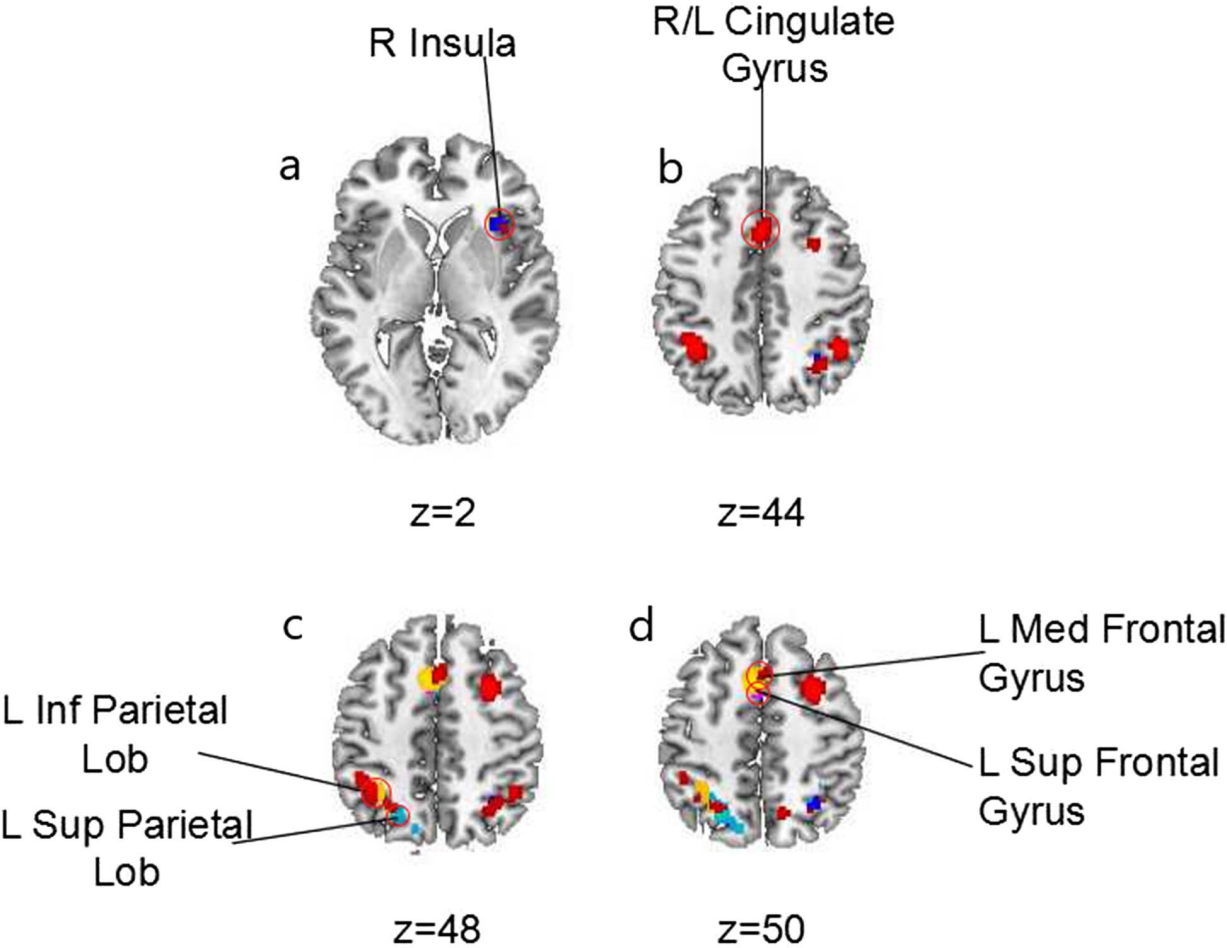
Significant overlay regions of 5 executive functioning tasks in included publications. Task 1 = n-back (yellow), Task 2 = reasoning / problem Solving (violet), Task 3 = task switching (light blue), Task 4 = Go/No-Go (red), Task 5 = visuospatial attention (blue). Imaging a, overlay region R Insula: center = (34, 22, 2), r = 8; Imaging b, overlay region Cingulate Gyrus: center = (2, 18, 40), r = 9; Imaging c, overlay region L Inf Parietal Lob: center = (− 36, − 48, 46), r = 10; L Sup Parietal Lob: center = (− 21, − 63, 51), r = 9; Imaging d, overlay region L Sup Frontal Gyrus: center = (− 2, 15, 50), r = 6; L Med Frontal Gyrus: center = (− 3, 9, 50), r = 6

We overlayed the 5 resulting task-specific maps, and projected them onto a brain template. The resulting overlay map is shown in Fig. 2. The results demonstrated that overlay regions of the 5 tasks included R Insula (BA13), Cingulate Gyrus (BA32), L Med Frontal Gyrus (BA32), L Sup Frontal Gyrus (BA6), L Inf Parietal Lobule (BA40), and L Sup Parietal Lobule (BA7). Hence, we confirmed that these 5 tasks can be combined into a meta-analysis of executive function.

### ALE meta-analysis results for change from pre- to post-CT

**Fig. 3:**
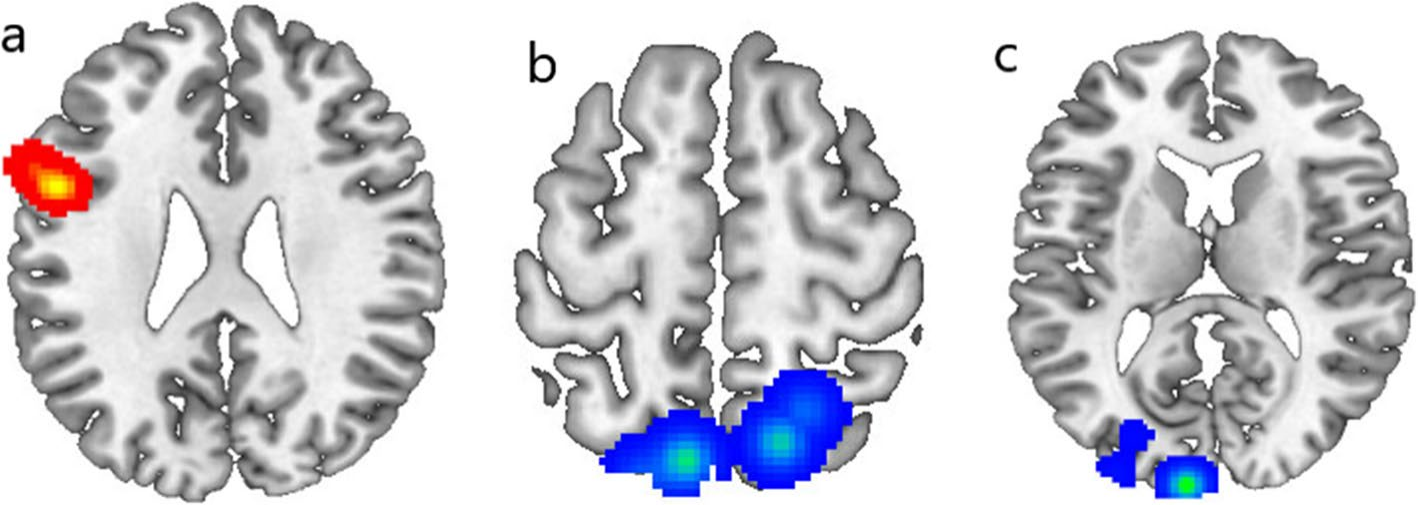
Areas of increased brain activation (red overlay) or decreased brain activation (blue overlay) after cognitive training

Two ALE meta-analysis were performed separately on brain areas displaying increased or decreased activation with *P* value = 0.05, cluster-level = 0.05 and threshold permutations =1000. The results showed that the left IFG (MNI -52, 8, 26; BA9) was significant for increased activation (maximum ALE value = 0.048), and the left precuneus (MNI -8, − 70, 60; BA7; maximum ALE value = 0.006) and the left cuneus (MNI -8, − 100, 12; BA17; maximum ALE value = 0.008) were significant for decreased activation. Results are shown in Fig. 3.

### Behavioral analysis of regions that reliably change from pre- to post-CT

**Fig. 4:**
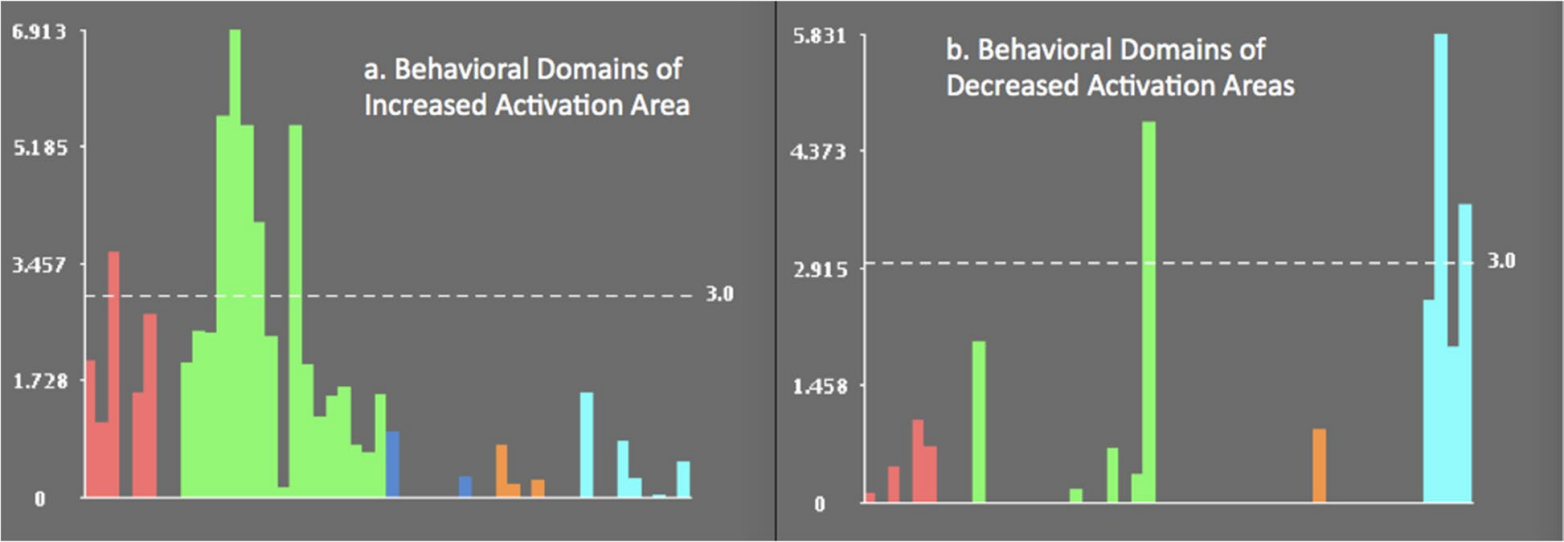
Histogram of the results of the behavioral analysis for regions implicated in CT. **a**. shows the behavioral domain Z-scores of regions of increased activation and **b**. shows the behavioral domain Z-scores of regions of decreased activation. Significant outcomes are on the dashed line. Blue: perception, Green: cognition, Red: action

As shown in Fig. 4, the left IFG ROI was significantly related to the cognition behavioral domain. In the cognition subdomain of language, there were several significant relations between the left IFG with semantics (*Z* = 6.913), phonology (*Z* = 5.659), speech (*Z* = 5.517), and syntax (*Z* = 4.07). The left IFG ROI was also significantly related to the memory subdomain of cognition (working) (*Z* = 5.51) and the action behavioral domain (imagination) (*Z* = 3.647). The left precuneus and cuneus ROIs were significantly related to the behavioral domains of perception including the subdomain of vision (motion) (*Z* = 5.831), (shape) (*Z* = 3.73), and the cognition behavioral domain (spatial) (*Z* = 4.757).

### Results of the functional Meta-analytic connectivity modeling analysis

**Fig. 5:**
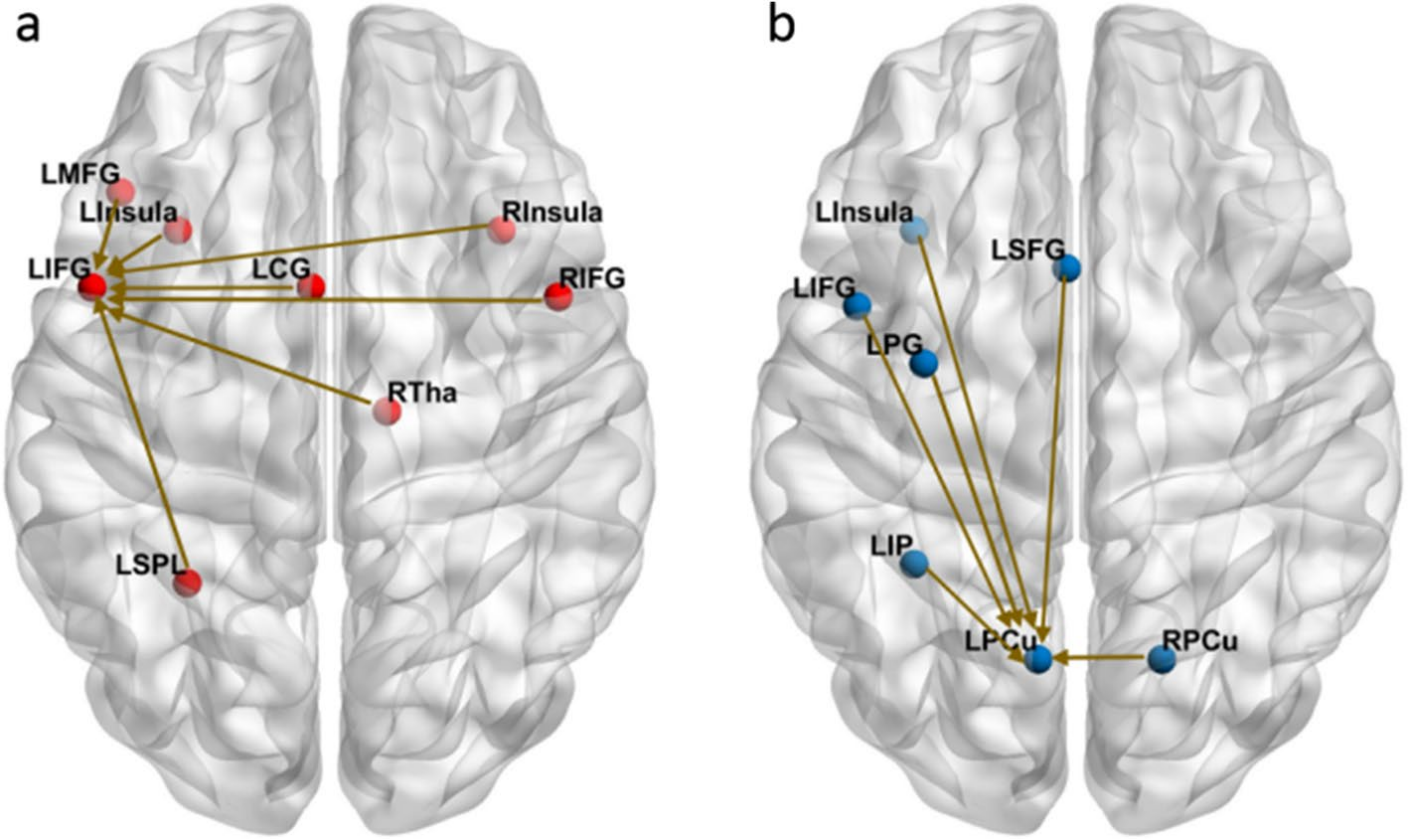
**a** The fMACA with left IFG as ROI, LIFG: left inferior frontal gyrus, LCG: left cingulate gyrus, LInsula: left insula, LSPL: left superior parietal lobule, LMFG: left middle frontal gyrus, RInsula: right insula, RIFG: right inferior frontal gyrus, RTha: right thalamus; **b** The fMACA with left precuneus as ROI, LPCu: left precuneus, LSFG: left superior frontal gyrus, RPCu: right precuneus, LIP: left inferior parietal lobule, LIFG: left inferior frontal gyrus, LInsula: left insula, LPG: left precentral gyrus

To find the task-dependent functional co-activations of the three regions altered by CT (left IFG, left precuneus, left cuneus), they were set as ROIs and three fMACM were constructed. For each region, we constructed fMACM that identified task-based co-activations across 73 neuroimaging studies of healthy participants (the least number of studies common to all 3 regions). These studies included 106 experiments with 842 subjects and 1631 locations for the left IFG analysis; 94 experiments with 1109 subjects and 1583 locations were used for left precuneus analysis; and 93 experiments with 1185 subjects and 1940 locations were used for left cuneus analysis. Results are shown in Fig. 5. The left IFG displayed significant co-activation with left cingulate gyrus (BA24), left insula (BA13), left superior parietal lobule (BA7), left middle frontal gyrus (BA46), right IFG (BA9), right insula (BA13), and right thalamus. The left precuneus demonstrated significant co-activation with left superior frontal gyrus (BA6), left IFG (BA6), right precuneus (BA7), left inferior parietal lobule (BA6), left insula (BA13), and left precentral gyrus (BA6). No significant co-activation was found between the left cuneus with other brain regions.

### fMACM between the 3 altered brain regions and the 5 executive task-activated regions

**Fig. 6:**
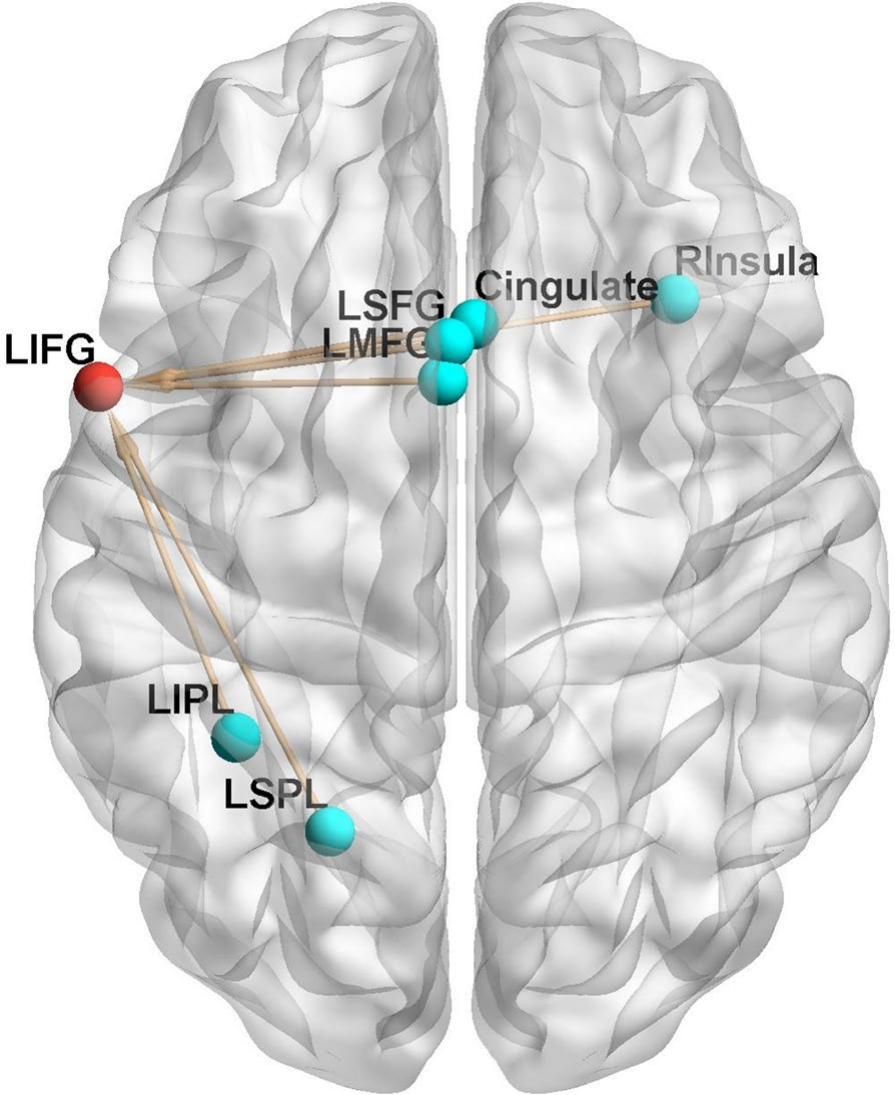
This figure shows the fMACM between the 3 brain regions altered by CT and the 5 executive task-activated regions. Red overlay = left IFG. Light blue overlay =5 executive task-activated ROIs, R Insula (34, 22, 2), Cingulate Gyrus (2, 18, 40), LIPL = L Inf Parietal Lobule (− 36, − 48, 46), LSPL = L Sup Parietal Lobule (− 21, − 63, 51), LSFG = L Sup Frontal Gyrus (− 2, 15, 50), LMFG = L Med Frontal Gyrus (− 3, 9, 50). Arrows are on behalf of co-activations in the fMACM with direction from activation ROI to co-activation overlay

Similarly, we constructed a fMACM between the 3 brain regions altered by CT and 5 executive task-activated regions. As mentioned above, the overlapping areas activated by the 5 executive tasks included R Insula, Cingulate Gyrus, L Med Frontal Gyrus, L Sup Frontal Gyrus, L Inf Parietal Lobule, and L Sup Parietal Lobule. We also analyzed the mean volume of 5 executive task-activated regions in the 3 seed regions (sphere, radius = 9 mm). The results showed all 5 executive task-activated regions were significantly related to the task-dependent functional coactivation regions of L Inf Frontal Gyrus (ALE value > 0.01). The results are shown in Fig. 6.

## Discussion

In this study, we conducted a meta-analysis to identify brain regions that are consistently reported in functional neuroimaging studies of CT for patients with psychiatric disorders. The main finding was that CT is associated with increases in activation in the left IFG and decreases in activation in left precuneus and cuneus from pre- to post- CT. This suggests that the response to brain activity may be a potential mechanism for improved cognitive function.

Generally speaking, decreasing activation putatively reflects reduced recruitment of neuronal resources, whereas increasing activation is thought to reflect the recruitment and evolution of additional neuronal substrates. Decreasing activation may presumably relate to the setting of more efficient task representations with repeated experience, whereas increased activation may be related to the establishment and development of task-specific representations with continued practice [25].

Behavioral analysis shows the left IFG is related to language, working memory, and imagination. Previous reviews and meta-analyses suggest the left IFG participates in semantic processing, phonology, syntactic processes, cognitive control, and working memory [25]. Some recent studies have also shown the left IFG regulates strategic semantic access via top-down signals acting upon temporal storage areas [26], and this function is affected by prior knowledge [27]. A recent meta-analysis of schizophrenia also found an increase activation in left IFG after cognitive training [14]. Cognitive training can improve the cognitive function of patients with bipolar disorder by increasing the resting cerebral blood flow in the left IFG [28]. Left IFG increased activation mainly improves verbal fluency and verbal working memory [29, 30]. All of this evidence supports the idea that left IFG is important for the core cognitive activity of language and working memory. Furthermore, this suggests that CT does indeed increase cognitive function through increased IFG activation.

Behavioral analysis of the left precuneus and left cuneus reveal that they are frequently activated while processing visual motion and shape. Research has proven that the precuneus is involved in a wide spectrum of highly integrated tasks including visuo-spatial imagery and episodic memory retrieval [31]. The cuneus, traditionally, has been linked to the processing of visual information, and also plays a supporting role in the integration of sensory information with cognitive processes such as attention, learning, and memory [32]. Studies indicate that the precuneus and cuneus are located in the default mode network (DMN) [33–37] where they play an important role [38, 39]. Precuneus and cuneus are related to cognitive dysfunction in patients with mental disorders [40, 41]. Decreased activation of left precuneus after cognitive training is associated with cognitive improvement [42]. Typically, relative DMN activity decreases during task performance [43], suggesting that CT improves cognitive function by reducing neurological resources for vision.

Furthermore, we conducted a fMACM to identify patterns of connectivity that have been frequently associated with the 3 seed regions (left IFG, left precuneus and left cuneus). We found that the left IFG was consistently co-activated with the left precuneus. This finding supports the notion that left precuneus reliably underlies visual processing for a wide spectrum of tasks and its activity supports the core cognitive function of left IFG. Consistent with the results of a recent study by our colleagues, we found that when performing a memory task, the bilateral anterior cingulate gyrus and precuneus are activated, while the left IFG and left cuneus are deactivated [44].

In the module of the left IFG, the left IFG and left superior parietal lobule belong to central-executive network (CEN), and the left IFG is one of its key nodes [45–47]. The CEN is involved in information manipulation and decision-making behaviors [46, 47]. Moreover, research has shown that during performance of cognitive tasks, the CEN typically displays increased activation [45, 48].

In the module of the left precuneus, the left precuneus, left superior frontal gyrus, and left inferior parietal lobule belong to DMN [45, 49]. The DMN is associated with emotional processing, self-referential mental activity, the recollection of prior experiences, and rumination [50–53]. As such, during performance of cognitive tasks, DMN is often deactivated; the DMN is “task negative”, i.e., it turns off when tasks are performed [43, 50, 54]. In psychiatric disorders, many studies have shown that poor cognitive performance is associated on an item-by-item basis (i.e., during event-related fMRI) with a failure to “turn down” the DMN [55–57]. That is, when the DMN is not shut off during task performance, the task is done poorly; when the DMN is shut off, the task is done well. As mentioned above, the left precuneus is a key node of DMN. This may mean that improvements in cognitive function are achieved by a rebalance within the DMN [58].

In addition, we can see that the left insula appears in both left IFG module and left precuneus module. The insula is implicated in disparate cognitive, affective, and regulatory functions, and it is a key node of the salience network (SN )[59, 60]. The function of SN is to segregate the most relevant among internal and extrapersonal stimuli in order to guide behavior [60]. We can speculate that SN plays a balanced role in both the left IFG and left precuneus fMACM modules. Meanwhile, previous studies have proposed that there is a complementary neurobiological relationship between the DMN and the CEN [45, 48, 52, 60, 61].

The results showed that cognitive training altered the activation of left IFG, left precuneus, and left cuneus. The 5 executive task overlay regions included R Insula, Cingulate Gyrus, L Inf Parietal Lobule, L Sup Parietal Lobule, L Sup Frontal Gyrus, and L Med Frontal Gyrus. We conducted a fMACM between the 3 brain regions altered by CT and the 5 executive task-activated regions and showed that all 5 executive task activation regions significantly coactivated with L Inf Frontal Gyrus. We noted that the brain regions altered by CT were not within the range of brain regions activated by the 5 executive tasks, but brain regions activated by the 5 executive tasks co-activated with L Inf Frontal Gyrus. This may indicate that changes in brain activation result from cognitive training rather than from the task itself, and associated changes lead to an improvement in cognitive function.

## Conclusions

Taken together within the context of our meta-analyses, we might conclude that CT improves cognitive function by supporting language and memory function, and reducing neuronal resources associated with basic visual processing. Perhaps the DMN and CEN play a role in balancing the neurological resources related to this change since CT helps “turn off” the DMN, allowing patients to turn their attention outward rather than inward.

### Limitations

There are several limitations of this study. First, the number of the included studies was comparatively small for ALE meta-analysis. Only 16 studies matched our screening criteria, which limits our power to detect consistently activated and deactivated regions. Secondly, we included 5 different executive function tasks in our screening criteria. Although we performed a validation and showed that the activation of the 5 tasks was overlapping, using a single task for meta-analysis improves accuracy. Thirdly, different mental disorders have different neuropathology that might affect the outcomes of CT, and for this study we combined several psychiatric disorders in order to have enough studies for the meta-analysis. Future studies will benefit from addressing these limitations.

## Data Availability

The data used for this meta-analysis are publicly available in the research studies. The full dataset can be requested from the corresponding author on reasonable request.

## Abbreviations

CT: Cognitive training
ALE: Activation likelihood estimation
fMACM: Functional meta-analytic connectivity model
IFG: Left inferior frontal gyrus
MNI: Montreal Neuroimaging Institute
MACM: Meta-analytic connectivity modeling
ASD: Autism spectrum disorder
SCZ: Schizophrenia
SAD: Schizoaffective disorder
ADHD: Attention Deficit/Hyperactivity Disorder
CRT: Cognitive remediation therapy
CR: Cognitive remediation
FAR: Facial Affect Recognition
DMN: Default mode network
CEN: Central-executive network
SN: Salience network
LIFG: Left inferior frontal gyrus
LCG: Left cingulate gyrus
LInsula: left insula
LSPL: Left superior parietal lob
LMFG: Left middle frontal gyrus
RInsula: Right insula
RIFG: Right inferior frontal gyrus
RTha: Right thalamus
LPCu: Left precuneus
LSFG: Left superior frontal gyrus
RPCu: Right precuneus
LIP: Left inferior parietal lobe
LInsula: Left insula
LPG: Left precentral gyrus
